# Freeze-Drying Process for the Fabrication of Collagen-Based Sponges as Medical Devices in Biomedical Engineering

**DOI:** 10.3390/ma16124425

**Published:** 2023-06-16

**Authors:** Chrysoula Katrilaka, Niki Karipidou, Nestor Petrou, Chris Manglaris, George Katrilakas, Anastasios Nektarios Tzavellas, Maria Pitou, Eleftherios E. Tsiridis, Theodora Choli-Papadopoulou, Amalia Aggeli

**Affiliations:** 1 Department of Chemical Engineering, School of Engineering, Aristotle University of Thessaloniki, University Campus, 54124 Thessaloniki, Greece; ckatrilak@cheng.auth.gr (C.K.); karipidn@cheng.auth.gr (N.K.); nestoraspetrou95@gmail.com (N.P.); cmanglar@ece.auth.gr (C.M.); katrilakas@gmail.com (G.K.); 2 3rd Department of Orthopedics, School of Medicine, Aristotle University of Thessaloniki, University Campus, 54124 Thessaloniki, Greece; tzav_a@hotmail.com (A.N.T.); etsiridis@auth.gr (E.E.T.); 3 School of Chemistry, Aristotle University of Thessaloniki, University Campus, 54124 Thessaloniki, Greece; pitoumaria91@gmail.com (M.P.); tcholi@chem.auth.gr (T.C.-P.)

**Keywords:** freeze drying, biomaterials, collagen, gelatin, alignment, medical devices, biomedical engineering, modeling, Artificial Intelligence

## Abstract

This paper presents a systematic review of a key sector of the much promising and rapidly evolving field of biomedical engineering, specifically on the fabrication of three-dimensional open, porous collagen-based medical devices, using the prominent freeze-drying process. Collagen and its derivatives are the most popular biopolymers in this field, as they constitute the main components of the extracellular matrix, and therefore exhibit desirable properties, such as biocompatibility and biodegradability, for in vivo applications. For this reason, freeze-dried collagen-based sponges with a wide variety of attributes can be produced and have already led to a wide range of successful commercial medical devices, chiefly for dental, orthopedic, hemostatic, and neuronal applications. However, collagen sponges display some vulnerabilities in other key properties, such as low mechanical strength and poor control of their internal architecture, and therefore many studies focus on the settlement of these defects, either by tampering with the steps of the freeze-drying process or by combining collagen with other additives. Furthermore, freeze drying is still considered a high-cost and time-consuming process that is often used in a non-optimized manner. By applying an interdisciplinary approach and combining advances in other technological fields, such as in statistical analysis, implementing the Design of Experiments, and Artificial Intelligence, the opportunity arises to further evolve this process in a sustainable and strategic manner, and optimize the resulting products as well as create new opportunities in this field.

## 1. Introduction

Thanks to significant advances in engineering, biology, and medicine over the past decades, biomedical engineering and its products, medical devices, have become a rapidly emerging field worldwide. Tissue engineering is a branch of biomedical engineering and is defined as an interdisciplinary area that combines engineering and the life sciences, in order to develop medical devices that can restore or replace damaged human tissues [1]. Currently, for example, considerable interest is observed in medical devices, such as implants, which can be used for hard tissue regeneration. Hard tissue engineering may be applied to bone defects such as those caused by operations, fractures, osteoarthritis, and osteoporosis, treated through the fabrication of implants, which should have some explicit characteristics [2]. For instance, their mechanical properties should be able to support and stimulate the formation of tissue; moreover, the engineered implant should have a pore size capable of allowing cell migration, the conveyance of nutrients, and angiogenesis, and it should have an appropriate biodegradation rate. To this end, biofabrication techniques, such as electrospinning, solvent casting/particle leaching, gas foaming, freeze drying, and 3D printing, are explored using a plethora of precursors, polymers, and composites [3,4,5,6]. A biofabrication process that is commonly applied to biopolymers found naturally in vivo, is freeze drying. Freeze drying is considered a mild process, usually carried out with water as a solvent which matches the hydrophilic properties of the in vivo biopolymers; furthermore, the lack of any other solvent or chemical during freeze drying can produce implants that have superior biocompatibility and preserved bioactivity. Collagen and its derivatives, such as gelatin, stand out among the many natural polymers that have been investigated for the biofabrication of implants, due to their key features, such as actual intrinsic in vivo biocompatibility, bioactivity, and biodegradability [2,7,8,9,10,11].

One of the most effective ways of developing collagen-based implants is by using the freeze-drying technology, which provides high-quality and shelf-stable implants under controlled conditions [12]. Furthermore, for the fabrication of this kind of medical device, a mild process such as freeze drying is needed, since collagen itself as well as additives, e.g., growth factors, may be sensitive to extreme conditions such as high temperatures and stresses. Freeze drying also ensures that the enclosed solvent is subtracted without substantial effects on the chemical properties of the protein or the risk of collapse of sensitive implant structures [13,14,15,16].

Additionally, the successful engineering of certain tissues, including muscle, neurons, ligaments, and tendons, depends on the macroscopic alignment of collagen implant structures. The fabrication of aligned collagen-based implants can be achieved by using a variety of processes including electrospinning, external electromagnetic fields, and mechanical forces. Electrospinning is a reasonably easy procedure that can quickly produce precisely aligned fibrous implants. However, it has been demonstrated that the flammable solvents, high shear, and strong electrical fields may result in toxicity or permanently disrupted collagen structure [17,18,19,20,21,22,23,24]. Alignment can also be induced during collagen self-assembly by introducing an external potential field, such as a magnetic field or an electrochemical pH gradient [18,25,26,27]. Finally, collagen fibers can be oriented by anisotropic strain towards the direction of the largest strain; the degree of strain anisotropy determines how strong the alignment is [18,28,29]. Nonetheless, these techniques call for highly specialized and frequently expensive equipment, making them challenging to scale up; an alternative emerging promising approach is directional freeze drying.

Thus, although freeze drying has traditionally been used by the food and pharmaceutical industries, this process is now also increasingly employed for the fabrication of collagen-based implants, and most notably collagen-based sponges, which are both scientifically and practically valuable. Highly open, porous three-dimensional dry biomaterials are employed for a wide range of applications in biomedical engineering [30,31]. The aim of this review is to present an overview of the basic principles, as well as new developments, opportunities, and challenges in the growing and rapidly changing field of the application of the freeze-drying process for the fabrication of collagen-based sponges in biomedical engineering applications.

## 2. Freeze Drying

**Figure 1:**
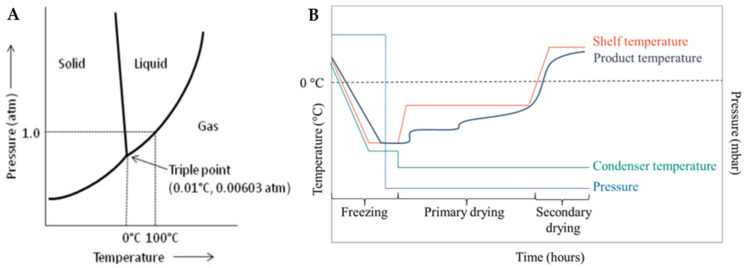
(**A**) Phase diagram of water [35] and (**B**) schematic diagram of the temperature and pressure changes during the freeze-drying process [36].

Freeze drying is often referred to as lyophilization, which means “loves the dry state”, although this phrase does not imply the freezing process. While the terms lyophilization and freeze drying are used as equivalent to each other, freeze drying seems to be more accurate. During this process, once the sample is completely frozen and placed at low pressure, the solvent, commonly distilled water, is sublimated, causing the ice to transition phase straight from solid to gas skipping the liquid phase, as shown in Figure 1A. Freeze drying involves three distinct and interconnected stages: freezing, primary drying (sublimation), and secondary drying (desorption) (Figure 1B). Each stage is based on different basic principles, with a significant impact on the final product [32,33,34].

### 2.1. Freezing

**Figure 2:**
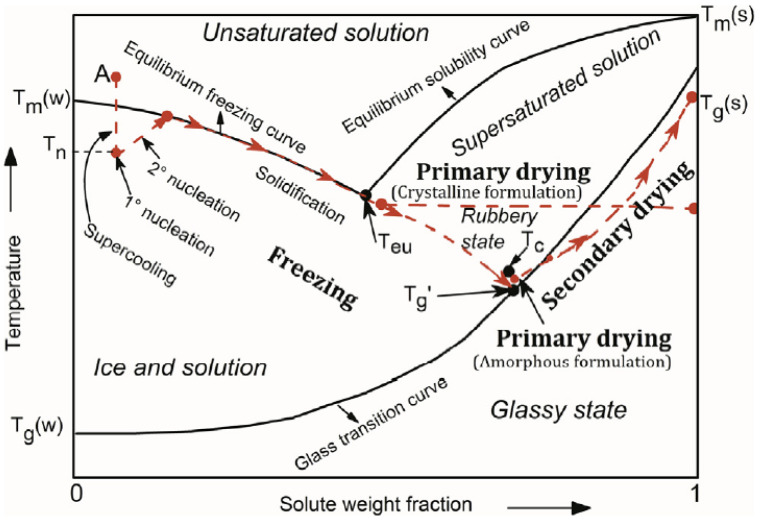
Phase diagram of a hypothetical solute-solvent system [37].

The initial phase, freezing, has a substantial impact on the overall performance of the freeze-drying process. In most cases, where the freeze-drying technique is used, the material that is being processed is not a pure solvent, but a multicomponent system. The existence of different components in the freezing mixture complicates the calculation and determination of its characteristic properties, such as the equilibrium freezing point, the eutectic point, and the glass and collapse temperatures, which are usually summarized in solute-solvent phase diagrams, such as the one presented in Figure 2 [12,34].

In order to initiate the freezing stage, the temperature of the system is lowered to its supercooling state, which is the difference in temperature between the equilibrium freezing point and the point at which ice nucleation initially develops in the solvent, known as ice nucleation temperature, T_N_ [12,38]. The degree of supercooling is determined by the physical parameters of the sample as well as the freezing method [39]. After the formation of the first ice nuclei, the solidification stage takes place, where the unbound water molecules become attached to the ice nuclei interface leading to the growth and therefore to the gradual increase in the solute concentration in between growing crystals in the formulation [37]; this may result in a profound restructuring of the internal architecture, especially of fragile materials, in certain cases giving rise to the characteristic lamellar structures often considered as the hallmark of the freeze-drying process. A type of material may not actually be completely frozen, although it might seem to be due to the amount of the existing ice; it is crucial to freeze the sample under the eutectic temperature, T_e_, so that no areas of unfrozen substance are left in the sample, and thus the product’s structural consistency that is freeze-dried is not jeopardized. T_e_ is the lowest temperature at which a solution remains liquid and below which the system is considered completely solidified in a crystalline structure. In amorphous systems, crystallization does not occur during freezing due to their complexity; however, as the solute weight fraction increases, the system becomes increasingly viscous reaching a glass-like state, as temperature drops [40]. The temperature at which this phenomenon occurs is known as the glass transition temperature, T_g_. T_g_ of a mixture is dependent on the water content; in general, the higher the water content before drying, the lower the glass transition temperature [38].

After the freezing stage, approximately 65–90% of the initial water content is frozen, with the rest remaining adsorbed. The freezing rate, ice nucleation temperature, and supercooling degree are all critical parameters determining the total drying time and the final properties of the product [38]. Rapid cooling produces ice crystals of smaller characteristic diameter, which are valuable for conserving microscopic structures but result in a material that is more challenging to freeze-dry. On the contrary, slower cooling results in bigger ice crystals and higher interconnectivity within the final matrix [33]. While there is no precise definition of slow and rapid freezing, a cooling rate of less than 1 °C/min is generally considered “slow freezing”, whereas a cooling rate of more than 100 °C/min is considered “rapid freezing”.

On the basis of the above information, it may be possible to envisage a number of process control parameters relevant to implant fabrication, such as gradual cooling, as mentioned above, or annealing the samples, in order to create fairly homogeneous ice crystals of a certain size. In some cases, the size of ice crystals may be controlled by annealing the frozen scaffold using freeze-drying cycles [41,42]. Annealing is the stage during which the material is kept for a controlled period at a point between the eutectic and the glass temperature in order to crystalize successfully. This approach has been found to stimulate ice crystal development and to speed up primary drying (the longest step in the process), enhancing overall the freeze-drying process [43].

### 2.2. Primary Drying

Once the molecules of free water are completely frozen, the next stage is primary drying. In this stage, the crystals of ice are removed through sublimation under vacuum conditions. In order to provide the latent heat for ice sublimation, the pressure is decreased to levels below the vapor pressure of water, while the shelf temperature is raised. When the temperature of the sample reaches the shelf temperature, and all of the unbound frozen water is completely sublimated, the primary drying stage is complete. In this way, after the removal of the ice crystals, an open network of pores is created, which supplies a route for the desorption of water from the sample throughout the next stage of the freeze-drying process [34,43].

The basic principle of primary drying is to choose an optimal product temperature, T_p_, quickly heat the sample to this temperature and maintain it at this constant during the primary drying process. For every product, during the primary drying stage, there is a critical temperature at which the product loses its macroscopic structure and collapses, namely, T_c_. As mentioned in the previous paragraph, the complete solidification of an eutectic system occurs below its eutectic point, which automatically makes T_e_ the maximum allowed product temperature, during primary drying. For an amorphous system, complete solidification occurs below T_g_, which is theoretically defined as the maximum allowed product temperature. Nevertheless, in most cases, it is observed that for amorphous systems, T_c_ differs from T_g_, and specifically is several degrees higher [37]. A higher product temperature results in a faster process. For every 1 °C rise in the product’s temperature, the primary drying time is reduced by around 13%, providing immense promise for reducing both the processing time and the production costs [32,43]. It becomes obvious that the optimization of primary drying is crucial because it occupies the majority of the freeze-drying cycle. Several time-consuming tests and research are required for the optimization of primary drying. In most cases, the optimization step is not performed and as a result, most materials are not freeze-dried under ideal conditions. In any case, the product temperature, which is dependent on the product’s composition features, the temperature of shelves, the pressure of the chamber, and the container system, cannot be precisely adjusted during primary drying and this lack of control is another major hurdle in the optimization of this process [44,45,46,47,48,49,50].

### 2.3. Secondary Drying

Secondary drying is the final stage of freeze drying, where most of the adsorbed or bound water that remains in the structure of the system is extracted via desorption. This process may begin during the primary drying stage, when ice crystals have sublimated in an area, creating a path for unfrozen water desorption. After primary drying, once all ice has been removed, the system still has a significant amount of residual water, accounting for 5 to 20% of the total initial moisture. In order for desorption to be achieved at a practical rate, the shelf temperature in the secondary drying stage is increased in comparison to that used for primary drying, following through the limitations mentioned in the previous paragraph, in order not to affect the structural stability of the material system during and after the secondary drying stage. The influence of residual water on the drying rate and the final drying time is remarkable. The time needed to eliminate the remaining water might be as much as, or even more than, the time required to remove free water [34,44,46,49].

Another feature of the desired final product that affects the adsorption–desorption equilibrium, and hence the secondary drying rate, is its specific surface area. As previously mentioned, fast freezing of samples results in the formation of many tiny crystals with a great surface area, which improves water desorption. On the contrary, and as it is expected, a slower freezing rate leads to a smaller surface area, which reduces the desorption of water, and as a result leads to a slower drying rate throughout this stage [38]. In any case, the aim of this stage is to reduce the residual water content to less than 1% [43].

### 2.4. Mathematical Modeling of the Freeze-Drying Process

The development of mathematical models of the freeze-drying process of biologically sensitive materials is of great value. Mostly, researchers focus on the mathematical description of the heat and mass transfer phenomena that take place during the drying stages of the process; however, the accurate measurement of the parameters and variables that are needed to solve these complex transport equations is extremely difficult. A number of publications regarding the mathematical modeling of the freeze-drying process have appeared in the last decades. At first, a more simplistic approach was followed, by applying steady state models [51], and later on by applying a more holistic approach featuring dynamic characteristics [52,53,54].

**Figure 3:**
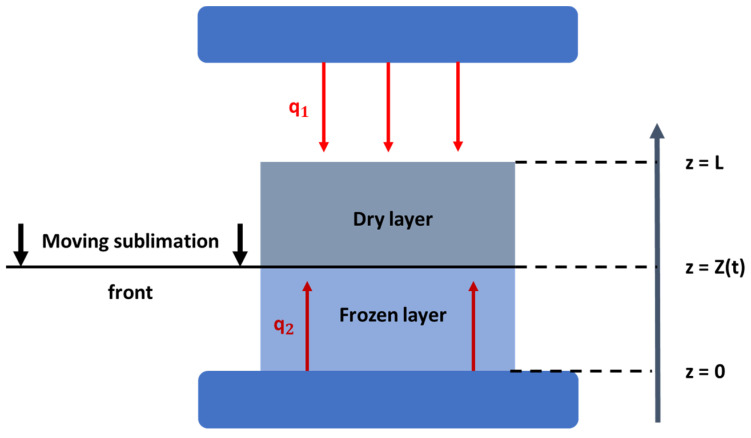
Schematic representation of the heat and mass transfer phenomena during the primary drying stage of the freeze-drying process [modified from [53]].

For the purposes of this review, a short indicative mathematical description of the process and the phenomena that take place during the primary drying process is mentioned as well as the assumptions that are made in most publications. As far as the process is concerned, in the freezing stage, the product becomes completely solid and then the pressure inside the chamber drops, in order for the rapid sublimation to be initiated. When sublimation begins, an interface between the dried and the frozen layer is created at the top of the product, which in most of the studies is assumed to be a uniformly retreating ice front, as presented in Figure 3 [51,54]. The retreat of this ice front continues until only a dry highly porous material is left behind, which signals the end of the primary drying stage of the process. Therefore, during this process, the upper surface of the product is heated via radiation that is released from the upper heating plate of the freeze-dryer setup, which is transferred through the gas phase and then by conduction through the porous material to the retreating ice front. At the other end of the material, heat is transferred by means of conduction through the frozen layer to the retreating ice front by the heating plate that is in direct contact with the material. Hence, when mathematical models are developed in order to describe the freeze-drying process, the heat and mass transfer equations have to be taken into account.

In most publications, a one-dimensional mathematical approach to the heat and mass transfer phenomena of the process is applied [52,53,54,55,56,57,58]. By making the assumptions that mass conservation takes place at the interface between the dried and frozen phase, that the same interface retreats evenly during the drying process until all free frozen solvent is removed from the material with a specific thickness, and that the heat transfer from the bottom of the shelf is carried out conductively, the equations that describe the phenomena take the following forms:

- Heat transfer in the frozen phase:

(1) $$\frac{{\partial T}_{f}}{\partial t}=a_{ef}\frac{\partial^{2}T_{f}}{{\partial z}^{2}}+\frac{Q_{vf}}{\rho_{ef}c_{pef}},\;0\leq z\leq Z\left(t\right),\;t>0,$$

- Heat transfer in the dried phase:

(2) $$\frac{{\partial T}_{d}}{\partial t}=a_{ed}\frac{\partial^{2}T_{d}}{{\partial z}^{2}}-\frac{c_{pg}}{\rho_{bud}c_{ped}}\frac{\partial \left(N_{w}T_{d}\right)}{\partial z}+\frac{Q_{vd}}{\rho_{bud}c_{ped}},\;Z\left(t\right)\leq z\leq L,\;t>0$$

- Mass transfer in the frozen phase:

(3) $$\lambda_{ef}\frac{{\partial T}_{f}}{\partial z}=\left(W_{p}-W_{eq}\right)\rho_{bud}{\Delta h}_{s}\frac{\partial Z_{f}}{\partial t},\;z=Z_{f},\;t>0$$

- Mass transfer in the dried phase:

(4) $$\lambda_{ed}\frac{{\partial T}_{d}}{\partial z}=-\left(W_{p}-W_{eq}\right)\rho_{bud}{\Delta h}_{s}\frac{\partial Z_{d}}{\partial t},\;z=Z_{d},\;t>0$$

where T_f_, T_d_ are the temperature of the frozen and dry layers, respectively; α_ef_, α_ed_ are the thermal diffusivity of the frozen and dry layers, respectively; z is the spatial coordinates; Q_vf_, Q_vd_ are the volumetric power of heat sources for the frozen and dry layers, respectively; ρ_ef_, ρ_bud_ are the density of the frozen and dry layers, respectively; c_pef_, c_ped_, c_pg_ are the specific heat of the frozen layer, the dry layer, and the water vapor, respectively; N_w_ is the flow of water vapor in the dry area; λ_ef_, λ_ed_ are the thermal conductivity of the frozen and dried layers, respectively; W_p_, W_eq_ are the initial moisture content and equilibrium moisture content, respectively; Δh_s_ is the enthalpy of sublimation; and Z_f_, Z_d_ are the sublimation front coordinates of the frozen and dry areas, respectively [54].

The kinetic characteristics of the primary drying stage, such as the variations in the product’s temperature and moisture content, can be calculated by solving the differential equations for the different phases. For example, some researchers tried to combine the mathematical models describing the primary drying stage of the freeze-drying process, utilizing a Quality by Design (QbD) approach, in order to create a well-defined design space [59]. In the QbD framework, the design space describes the multidimensional combination and interaction of input parameters that seem to ensure the quality of the final product. Moreover, in another research paper, scientists used the same combination of mathematical models and the QbD approach in order to scale up the primary stage of the cycle for the production of pharmaceutical products [60]. Therefore, the effect of a wide regime of different operating conditions on the final product can be a priori calculated and then validated experimentally, and as a result, the required drying time, energy, and financial costs can be reduced.

Freeze drying tends to preserve the biological activity of thermosensitive components better than the classical thermal drying methods, and in addition to that, the shelf life, storage, and transportation of the lyophilized products become optimized [55]. However, as a process, it also has some disadvantages, such as high time and energy consumption and high technological complexity [61,62]. Moreover, it makes the quantitative measurement of its characteristics, such as the temperature and moisture content of the product, as well as the distribution of pressure inside the chamber in the primary and secondary drying stages of the process, extremely difficult [63].

## 3. Freeze-Dried Collagen-Based Sponges in Biomedical Engineering

In the field of biomedical engineering, the fabrication of appropriate implants is considered to be one of the most important factors for the engineering of tissues [64,65]. It is of major importance that the structural, mechanical, and functional properties of the fabricated implants are compatible with those of the original tissue. Moreover, the implants should exhibit other desired properties, such as biocompatibility and biodegradability, as well as the ability to promote cell adhesion, cell proliferation, and differentiation for the formation of new tissue [66,67]. Cells are able to recognize the texture and arrangement of the implant; therefore, even topographical anisotropies have to be considered [68]. Freeze drying is one of the main available processes for the development of medical devices in the form of collagen-based, sponge-like implants for tissue regeneration, such as bone, muscle, cartilage, skin, and nerve tissue [41,69,70,71,72].

Collagen is one of the most used biomaterials for the fabrication of freeze-dried sponges in medicine [73]. Collagen is the most abundant protein in mammals and the main structural protein in the extracellular matrix (ECM), mainly found in connective tissues such as cartilage, bones, tendons, and ligaments, providing elasticity, stability, and support to the tissues and participating in cell signaling and migration [72,74,75,76,77,78]. In fact, approximately 25% of the whole-body protein content is collagen. Until now, 29 types of collagen proteins have been described which differ with respect to amino acid sequence, location in the tissues, and biological role, with type I collagen being the most prevalent in the body. The repetitive primary structure units are mainly [Gly-X-Y-]n, where Gly is glycine and X and Y are most frequently proline and hydroxyproline residues [79,80]. Collagen molecular weight varies depending on the type, with a typical value of ca 300 kDa, which tends to self-assemble into triple helical configurations forming nanofibrils with typical dimensions of 300 nm in length and 1.5 nm in diameter [81].

Another relevant, collagen-based, well-known biomaterial for the production of freeze-dried sponges for tissue regeneration is gelatin [72,82,83,84,85,86,87,88]. Gelatin is a heterogeneous mixture of water-soluble proteins originating from collagen that is commonly used in medical applications due to its biocompatibility and biodegradation [84,85,87,89]. It can be sourced from various mammals, mainly pigs, and cattle, and importantly it has been classified as a generally-regarded-as-safe (GRAS) substance by the Food and Drug Administration (FDA) [90,91]. Upon hydrolysis of collagen, the quaternary structure of its triple helix breaks into single, double, and triple chains, which retain the characteristic repetitive units of amino acids Gly-X-Y. Based on the type of hydrolysis process, acidic or basic, two gelatin types can be obtained. More specifically, type A gelatin derives from acid-cured (acid-hydrolyzed) tissue, while type B gelatin derives from lime-cured (base/alkaline-hydrolyzed) tissue. Their main differences are their charge and isoelectric point. Type A gelatin is positively charged at a neutral pH since it has an isoelectric point between 8 and 9, unlike type B gelatin which is negatively charged at a neutral pH and has an isoelectric point between 4.8 and 5.4 [85,92]. Due to its biocompatibility, bioactivity, and biodegradability along with its low cost and absence of antigenicity, collagen as well as gelatin are considered top-choice materials in biomedical engineering, an increasingly popular application being collagen-based sponges as medical devices [3,41,93].

Despite the many advantages of collagen-based sponges, some challenges concerning their properties have also been recognized that merit optimization. In some tissue engineering studies, they have shown low mechanical strength compared to the tissue requirements, combined with a high degradation rate [18]. As a result, they may fail to induce successful tissue repair [94]. Furthermore, although collagen-based sponges have an inherent ability to promote cell attachment and proliferation, in some circumstances, functional molecules, such as growth factors, should be included in the sponge in order to boost the regeneration potential. Concerning these functional molecules, an important challenge faced by scientists is the type of the biomolecule, its concentration depending on the target tissue, and the release rate. Collagen has a low affinity for some growth factors which may result in poor sustained and slow release needed for tissue repair applications. In addition, the incorporation of growth factors increases clinical costs and hospital charges [95,96]. In other studies, adverse effects have been reported due to poor growth factor control by the collagen-based sponge; for instance, in osteogenesis where collagen sponges are used as carriers of recombinant human Bone Morphogenetic Protein-2 (rhBMP-2), burst release and leakage in vivo can result in an unexpected local increase in BMP-2, which is linked to adverse effects, i.e., postoperative inflammation, ectopic bone formation, bone resorption, and at a low percentage, even cancer [96,97].

### 3.1. Parameters Affecting the Properties of Collagen-Based Sponges

In order to achieve the desired outcome in biomedical applications, the control of the mechanical and physicochemical properties of the fabricated sponge has been an area of great interest in the scientific community over the past decades [88,98]. One approach to controlling and finally fabricating the desired structure of the sponge involves alternating the freeze-drying process variables, such as the freezing rate of the sample, the applied pressure in the chamber, the temperature, and the time under which the sample is processed, as well as the presence or absence of mold. For example, some scientists studied how the variations in the cryogenic parameters applied before the primary drying of the process, as well as the variations in freezing temperatures, freezing rates, and freezing methods, enable the development of sponges with different pore morphologies [69,99]. Others investigated how a controlled rate of freezing can affect the reproducibility of the microstructure of the fabricated sponge [100,101], and others performed a more holistic investigation by combining different parameters of the whole process, such as freezing temperature, type of mold, and concentration of the solvent, with the mechanical and physicochemical properties of the freeze-dried sponge [9,70,71,76,102].

**Figure 4:**
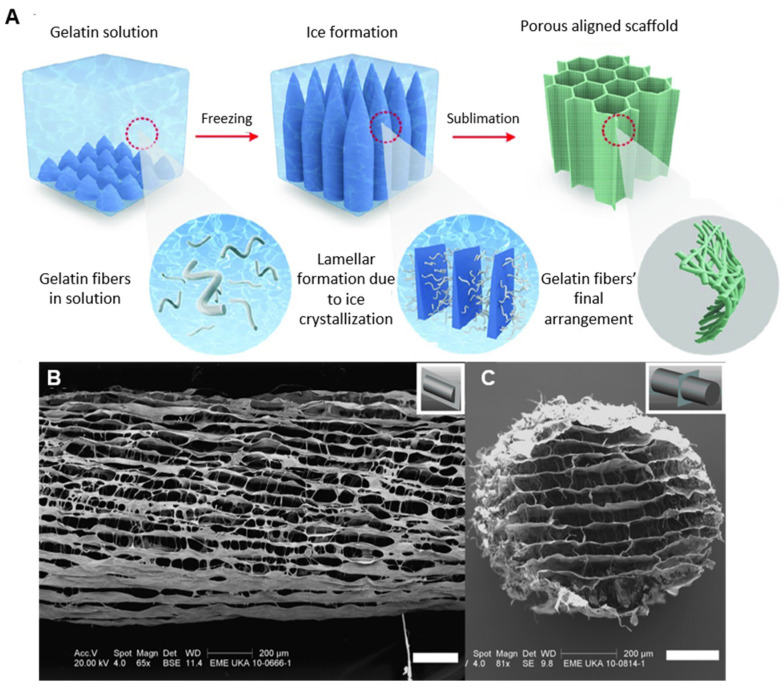
(**A**) Schematic representation of the process for the fabrication of aligned collagen-based sponges [modified from [134]], (**B**) longitudinal microstructure of an aligned collagen-based nerve guide, and (**C**) transverse microstructure of an aligned collagen-based nerve guide (Scale bars: **B**,**C** = 200 μm) [24].

Regarding the fabrication of macroscopically aligned collagen-based sponges, especially important for skeletal, muscle, and nerve tissue engineering, this may be achieved by means of directional freeze drying [103]. The key principle here is to establish a temperature gradient along a specific direction of the material during freezing, usually by employing a specially designed material holder that has insulating and non-insulating sides; the insulating sides do not allow the material to feel the temperature of the freezing medium, whilst the non-insulating sides do. In this way, the ice crystals grow in one direction in the design (Figure 4) [24,104,105,106,107,108,109,110,111,112,113,114,115,116,117,118,119,120,121,122,123,124]. After the completion of the freeze-drying process, an anisotropic lamellar structure is obtained. This process can be used to produce collagen-based sponges with anisotropic properties, where the properties of the material differ depending on the direction in which they are measured [125,126]. However, these sponges tend to have an especially delicate structure and they are prone to collapse [126,127,128,129]. An example of the final structure of a collagen-based sponge produced by this type of technique can be seen in Figure 4 [130,131,132,133,134].

At the same time, in order to enhance the biological, physical, and chemical properties of the sponge, the main collagen/gelatin ingredient is often combined with additives and crosslinkers [135]. Scientists investigated different approaches; some focused their research on the addition of different additives to collagen-based sponges, in order to examine how various additives’ contents affect the morphology, the physicochemical characteristics, the compressive mechanical properties, and the cytocompatibility of the sponge [101,136,137]. Others investigated the effects of the type of collagen, its concentration, and its molecular weight on the properties of the fabricated sponges [41,69,102,138,139]. Last, but not least, it has also been observed that the cross-linker concentration, crosslinking method, and exposure time have a significant effect on the final sponge properties [41,102,140,141]. Several crosslinking techniques have been developed, which are divided into physical, chemical, and enzymatic techniques [72,88]. For instance, physical crosslinking includes dehydrothermal treatment (DHT) and UV irradiation [142]. Some of the most used chemical crosslinkers are 1,4-butanediol diglycidyl ether (BDDGE), glutaraldehyde (GA), and 1-Ethyl-3-(3-dimethylaminopropyl)carbodiimide (EDC) coupled with N-hydroxysuccinimide (NHS) and genipin (GNP), while enzymatic crosslinking is performed with the transglutaminases, which are enzymes found throughout the body that create bonds among the collagen’s monomers, leading to even higher molecular weight molecules [41,143].

**Figure 5:**
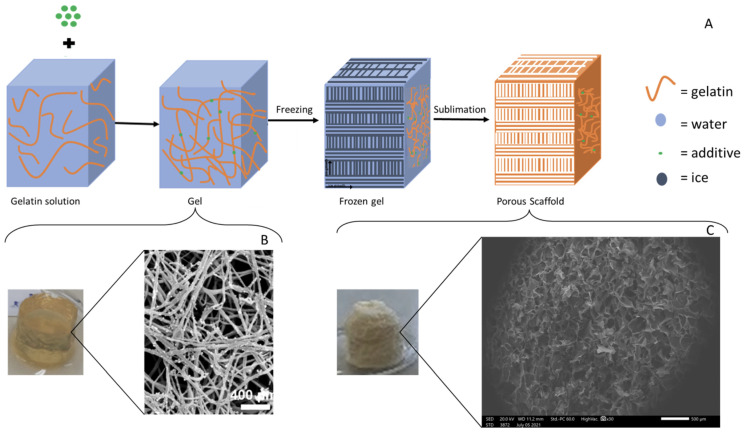
(**A**) Generic schematic representation of the beginning-to-end fabrication process for the collagen-based-additive freeze-dried sponges, (**B**) macro- and microstructure of the starting hydrogel, and (**C**) macro- and microstructure of the final freeze-dried sponge. Scale bars: **B** = 400 μm and **C** = 500 μm [**B**,**C** modified from [166]].

In order to satisfy the requirements of the intended application, the properties of the resulting collagen-based sponge may be tuned using various additives, which can be categorized based on their chemical composition and properties. Some well-studied inorganic additives are hydroxyapatite (HAp) and bioactive glass (BG), while chitosan (CH), silk fibroin (SF), cellulose (CE), glycosaminoglycan (GAG), and hyaluronic acid (HA) are the most commonly used biopolymers. Additionally, synthetic polymers such as polylactic acid (PLA), tetracycline hydrochloride (TCH), polycaprolactone (PCL), and poly(lactic-co-glycolic acid (PLGA) [78,144] may be added to the starting collagen or gelatin solution, leading to a composite microstructure hydrogel before the freeze-drying process, as presented in the schematic in Figure 5. The inclusion of additives to the starting solution alternates the properties of the final scaffold; for example, the addition of biopolymers, such as chitosan, leads to a more homogeneous structure with smaller pore sizes, while the use of synthetic polymers, such as PLA reinforces the collagen-based sponges and in some cases, improves their thermal stability [69,145]. In hard tissue engineering, researchers presented freeze-dried collagen-based sponges in conjunction with the inorganic additives of hydroxyapatite or bioactive glass that stiffen the scaffolds up to 10–20 times, to better fit the requirements of bone regeneration [93,146,147,148,149,150,151,152,153,154,155,156,157]. In some cases, these were also enhanced by the addition of biopolymers, such as glycosaminoglycan, aiming to induce the vascularization of tissue-engineered implants [158,159,160]. Moreover, freeze-dried sponges with suitable pore sizes are frequently used in cartilage regeneration; PLGA-collagen hybrid sponges are considered especially promising for such applications [78,161,162,163]. Another well-known use of these sponges, this time with additives such as PLA, polyvinyl alcohol (PVA), and chitosan, is for skin tissue engineering purposes, such as wound healing [145,164,165].

The key additives, the crosslinking methods, and the freeze-drying operating parameters mainly used to prepare collagen-based sponges are summarized in Table 1. It must be noted that by controlling the various parameters affecting the fabrication process, a Quality by Design (QbD) approach can be achieved, meaning that researchers can understand the effect of each parameter on the final scaffold and design the fabrication process based on their needs.

### 3.2. Commercially Available Collagen-Based Sponges

The development of collagen-based sponges through the freeze-drying process resulted in the commercial availability of these novel medical devices for a variety of biomedical engineering applications, mainly dental, orthopedic, hemostatic, and neuronal. For bone regeneration, commonly in cases of spinal fusion, tibial fractures, and maxillofacial grafts, INFUSE^®^ Bone Graft and InductOs^®^ produced by Medtronic BioPharma B.V. are used, which are identical products manufactured in the United States (approved by the FDA) and Europe (approved by the European Medicines Agency (EMA)), respectively [173,174]. Both of these commercial products consist of an active biological substance, rhBMP-2, in powder form, a solvent, and a collagen-based sponge fabricated using freeze drying [175]. It must be noted that these bone grafts exhibit side effects, such as ectopic bone formation and infections. Moreover, the clinical usage of rhBMP-2 has recently sparked controversy related to the way this protein is administered by the sponge, and to the quantity which is needed to be added to it [176,177]. Despite the disadvantages, the benefits of these products are considered to outweigh their negative effects, leading them to be certified for use with generally positive clinical feedback [175,178].

**Figure 6:**
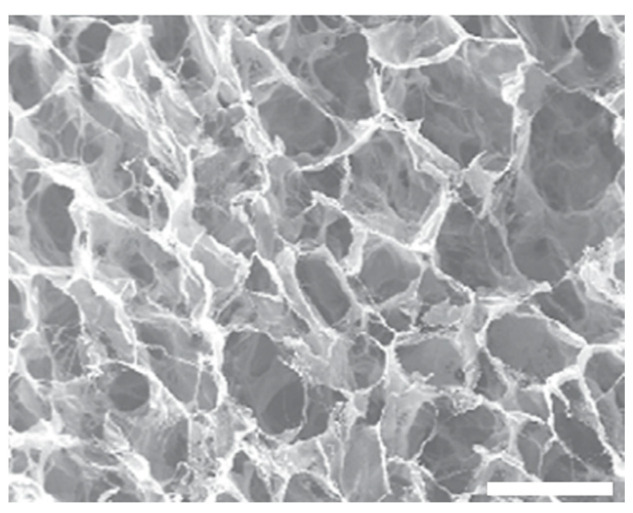
Scanning Electron Microscopy image of HELISTAT^®^ (Scale bar: ~200 μm) [182].

The collagen-based sponge of the above-mentioned products is HELISTAT^®^ produced by Integra LifeSciences Corporation, which is commonly used as a hemostatic agent but has also been employed as a carrier for the delivery of proteins, such as BMPs, due to its 3D and porous structure. HELISTAT^®^ is a soft, white, flexible, and absorbent sponge derived from collagen obtained from the Achilles tendon of cattle, which makes the final product highly stable due to its purity [179,180,181,182]. The porous structure of the freeze-dried collagen sponge HELISTAT^®^ is presented in Figure 6.

Another well-known commercial sponge for nerve tissue engineering is NeuraGen^®^ produced by Integra LifeSciences. It is a 3D Nerve Guide Matrix specifically designed to heal peripheral nerve discontinuities and increase the functional regeneration of wounded nerves across 5 to 15 mm gaps, with substantial axonal regeneration at 2 weeks post-implantation. It is fabricated by type I bovine collagen and has two collagen structures, the outer and inner, which are both freeze-dried. The outer collagen structure provides a semi-permeable, porous barrier that separates and guards the peripheral nerve while axons regrow. Additionally, it retains the Nerve Growth Factor and permits the diffusion of tiny nutrient molecules. The inner collagen matrix, on the other hand, is a distinctive, porous matrix where chondroitin-6-sulfate (glycosaminoglycan) is infused and longitudinally aligned to promote cell proliferation. NeuraGen^®^ guides, as demonstrated in numerous clinical studies, have a strong affinity for regenerating cells and may be applied as an effective cell delivery system [183,184,185,186,187,188,189,190].

**Figure 7:**
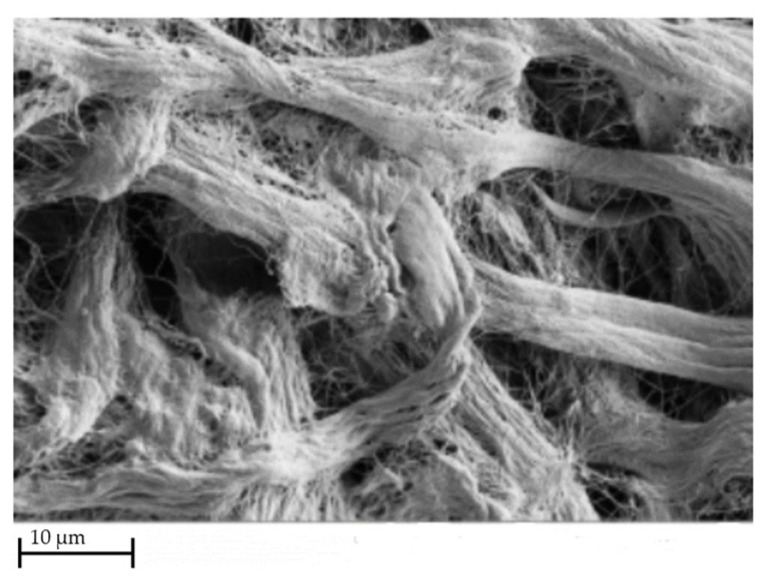
SEM image of GraftJacket™ matrix [192].

In addition, it must be noted that commercially available skin substitute products exist, some that derive from the human dermis, such as GraftJacket^™^ matrix (Wright Medical Group N.V., Memphis, TN, USA) and Coll-e-derm^™^ (Parametrics Medical, Leander, TX, USA), and others that derive from animal tissues, such as Architect^®^ (Harbor MedTech, Inc., Irvine, CA, USA). These products undergo a specific process that renders the collagenous material acellular (decellularization process) and then they are freeze-dried in order to preserve the matrix; a Scanning Electron Microscopy (SEM) image of the GraftJacket™ matrix is presented in Figure 7. They have desirable scaffold characteristics, such as biocompatibility, minimizing the inflammation phase, and suitable mechanical characteristics that mimic the native tissue, which preserve cell signaling factors to trigger and accelerate healing and tissue regrowth [191].

In addition, freeze-dried collagen sponges are offered commercially as wound dressings for tissue regeneration. Namely, Ologen™ Collagen Matrix (Aeon Astron Europe B.V.) is a medical device specifically designed for ophthalmic surgeries, such as glaucoma surgery. It is made from a porous matrix of crosslinked atelocollagen derived from the skin of cows and has less than 10% of glycosaminoglycan in order to promote fibroblast ingrowth for wound healing. Its three-dimensional structure supports the connective tissue and is important for the maintenance of its elasticity and strength [191].

Commercially available freeze-dried collagen scaffolds are also commonly found in the field of dentistry. Mucograft^®^ (Geistlich Pharma AG) is a pure collagen type I and III non-crosslinked membrane, obtained by a standardized controlled manufacturing process, and sterilized by means of gamma irradiation. It has a bilayer structure with one smooth, non-permeable layer and one porous one, which consists of collagen fibers in a loose arrangement to encourage tissue regrowth. Moreover, due to their biocompatibility and biodegradability, collagen membranes are utilized for Guided Tissue Regeneration and Guided Bone Regeneration. The fundamental idea of these procedures is the application of a barrier membrane to distinguish between slow-proliferating regenerative cell types, such as osteoblasts and periodontal cells, and fast-proliferating epithelial and connective tissue cells, allowing the regeneration of the lost tissue. Jason^®^ and collprotect^®^ fabricated by Botiss Biomaterials, a biotech company from Germany, are a non-crosslinked and a crosslinked membrane, respectively, derived from porcine tissues [193]. The Jason^®^ membrane derives from a dense collagenous structure, resulting in a rigid and tear-resistant membrane that undergoes slow enzymatic degradation, providing an extended barrier time for the treatment of larger defects. On the other hand, the collprotect^®^ membrane has a dense but open, porous collagen structure and it is used for dental bone and tissue regeneration. The commercial products mentioned in this paragraph exhibit the characteristic lamellar structure under the scanning electron microscope, which indicates that they are likely fabricated using freeze-drying technology; however, there is scarce information in the literature to establish this.

## 4. Design of Experiments and Artificial Intelligence

**Figure 8:**
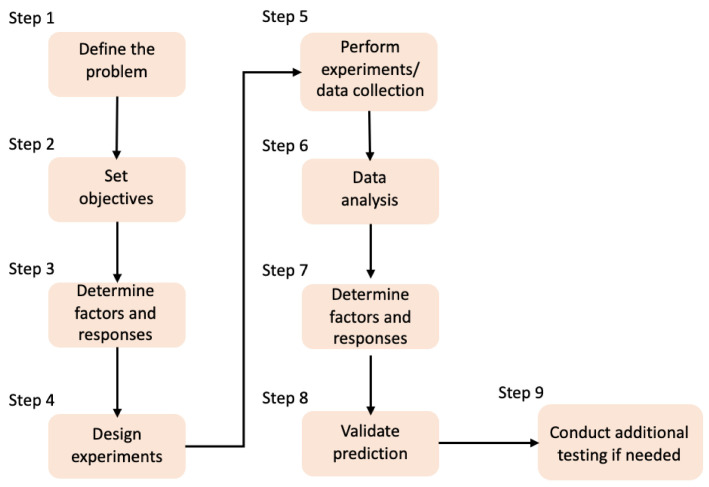
DoE workflow for the process optimization [194].

Nowadays, the use of technology for the replacement of human effort and time as well as the reduction in material resources has become an essential need. Lab scientists can use the power of an artificial brain in order to test their hypothesis and speed up and verify the experimental procedure. In that direction, several Design of Experiments (DoE) methods are being used widely to optimize the processes by reducing the running time and the experimental costs. In Figure 8, a DoE workflow is presented for process optimization. Nevertheless, DoE is a human-centered method, depending on the researcher’s knowledge of the process, that actually selects the input factors to be included in an experiment. On the other hand, in the past decades, another automated process has been developed, where data patterns are detected, based on both the input and the output data [194,195]. Additionally, the classical laboratory approach is supported by Data Science, and it combines human knowledge, statistics, and artificial knowledge, thus producing faster and more reliable results. According to Rebala G. et al., “Machine Learning (ML) is a field of computer science that studies algorithms and techniques for automating solutions to complex problems that are hard to program using conventional programming methods.”, whereas Artificial Intelligence (AI) refers to the concept of creating intelligent machines [196].

Basically, there are two approaches that can be used in order to apply ML to the subject of this review. The first would be to use Classical ML algorithms, which are more human-interpretable statistical methods, wherein the scientist can draw conclusions about the data themselves and validate a hypothesis. These classical approaches include classification, Regression, and Clustering Algorithms. The first two are under the Supervised Learning (SL) and the third one is under the Unsupervised Learning (UL) models. For the SL algorithms, the machine needs human input in order to validate the results or provide material in order for the model to learn. Thus, the scientists can either come across some features or combinations that can improve their results or make the predictive procedure faster (depending on the “question”). As for the UL model, its method is based on “digging out” common patterns. This means that the data may have underlying connections that are not “visible” and the UL algorithms can help in order to expose the “secret” message behind them.

Moving forward, the second approach would be the use of Deep Learning (DL). Bengio, Y. et al. defined the deeper level of ML known as DL, explaining the capabilities and end-use of Deep Neural Networks (DNNs). Specifically, he defines them as “architectures which are able to learn multiple levels of abstraction and representational power, enabling them to perform very well on a wide range of problems”. DNNs have exhibited outstanding performance in problem domains such as image and decision-making domains, which are common problem domains in the biomedical research field [197]. The NNs, as mentioned above, are inspired by the human brain and their functionality is a “black box” to the user. DL is used in complex recommendation systems or classification problems but the results are not easily explained by the user. The scientist can tune the hyperparameters of the models but it is not clear how these changes affect the network.

To further zoom in on the problem, there are several algorithms that can be used in order to provide insights into a study. Some simple techniques are decision trees or regression models. However, there are also more enhanced algorithms such as Random Forests, which use multiple decision trees and choose the best “solution” and ensemble pipelines which combine clustering and classifications.

To the best of our knowledge, Random Forests and NN regressors are mostly used to approach the characterization of the freeze-drying process. Specifically, in order to monitor the vacuum freeze-drying process of the desired product, a group of scientists used an object detector network based on a Faster Region Convolutional Neural Network (FasterR-CNN), combined with a Kernelized Correlation Filter (KCF) tracker [198]. Others investigated the effects of the microstructure of the sponge on the combination of various parameters, such as the drying conditions and solute environment. The impact that the drying temperature and the pressure of the chamber have on the pore wall roughness was revealed by the qualitative assessment of the experimental data, and additionally the influence that the collagen concentration, the solvent type, and solute addition have on the pore morphology was confirmed. To demonstrate quantitative differences, Random Forest Regression (RFR) was implemented to investigate multidimensional biometric data and predict the scaffold’s microstructural attributes. Regression models helped to evaluate the relative impact of the experimental parameters on pore analyses. Importantly, by using this approach, it was possible to identify techniques that can provide new valuable input information to the algorithms, with predictive power regarding the sponge features. Thus, this paper demonstrates the potential for predictive models such as RFRs to discover novel relationships in biomaterial datasets [199].

Based on these first attempts and their remarkable results, it seems that Artificial Intelligence is a powerful tool for the mathematical modeling of the freeze-drying process. It is able to shed light on the secrets behind the parameters affecting the outcome of the process and simplify the researchers’ analyses. However, it is noted that, currently, only a few studies mention its use in relation to this fabrication process.

## 5. Discussion/Conclusions

Biomedical engineering and its products, namely, medical devices, have become a rapidly emerging field worldwide thanks to significant advances in engineering, biology, and medicine over the past decade. Advances in biomedical engineering are largely correlated with advances in biofabrication techniques of medical devices. A biofabrication process that is commonly applied to biopolymers found naturally in vivo is freeze drying. Collagen and its derivatives, such as gelatin, stand out among the many natural polymers that have been investigated for the biofabrication of promising medical devices, such as open porous collagen-based scaffolds in the form of macroscopic sponges. Through the freeze-drying process, high-quality collagen-based biological substitutes can be developed with a multitude of applications as medical devices in biomedical engineering. Freeze drying allows in vivo biopolymers, mainly collagen, to form open porous three-dimensional sponges, with macroscopic internal alignment, when necessary, under controlled conditions, mimicking the structural, mechanical, and biological properties of the native tissue with the potential to act as an ideal scaffold in various hard and soft tissue engineering applications for the restoration or replacement of damaged human tissues. This fruitful research has already realized some of its potential uses by having been translated into a number of successful commercial medical devices, foremostly for dental, orthopedic, hemostatic, and neuronal applications.

However, it is noted that there are a number of open and pressing challenges still in this field. The freeze drying of collagen-based sponges is still considered a high-cost and time-consuming process that is often used in a non-optimized manner regarding both sustainability and final product features. Furthermore, it is often the case that the primary research results are reported in an unclear manner, creating confusion regarding the parameters that have actually been used during the process. Moreover, thorough characterization of the freeze-dried collagen-based sponges is quite rare. The most frequently reported features tend to be average pore sizes and, in some cases, the mechanical characterization of the sponges, whilst essential attributes such as lifetime and biodegradation in vivo, cell migration as well as angiogenesis tend to be studied and reported very rarely. The above omissions make it difficult to create sufficiently large datasets for further analysis and optimization.

**Figure 9:**
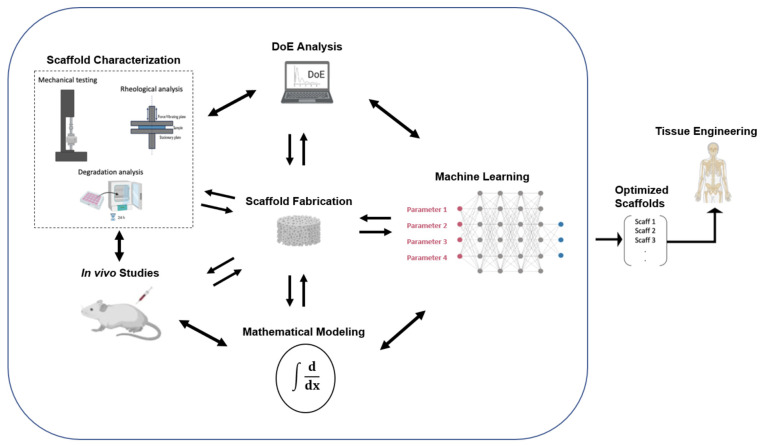
Schematic representation of the proposed optimization process for the fabrication of collagen-based sponges as medical devices in biomedical engineering [modified from [194]].

By combining interdisciplinary advances in other technological fields, the opportunity arises to further evolve this process, minimize the required lab time and resources, and simplify, or even optimize, the resulting products. In the near future, the researchers of this interdisciplinary field would have to not only familiarize themselves with the implementation of neuronal networks in their scientific work in order to achieve optimal results but also interactively combine them with DoE analysis, mathematical modeling, characterization techniques, and in vivo studies, as presented in Figure 9, aiming to enhance their research and their understanding of the freeze-drying process for the sustainable fabrication of optimized collagen-based sponges as medical devices in biomedical engineering.

## Figures and Tables

**Table 1:** Fabrication parameters of freeze-dried collagen-based sponges.

Type	Percentage	Additive	Crosslinking Method	Freezing Conditions	Freeze-Drying Conditions	Product Properties	Application	Ref.
Type I collagen from porcine dermis	-	HAp	GA	−20 °C (12 h)	24 h at −40 °C	50–100 μm, Homogenous pore structure, Stresses: up to ~3 MPa	Bone Tissue Engineering	[147]
Type I collagen from bovine	0.5% (*w*/*v*)	nano-HAp	DHT	−40 °C	17 h at 0 °C	porosity >98.9%, Homogenous pore structure, Compressive modulus: up to ~0.3 kPa	Bone Tissue Engineering	[148]
Type I collagen from bovine dermis	50 mg/mL	HAp whiskers	DHT	−80 °C	24 h at −30 °C	porosity 90−96% 20–90 μm, Elongated linear pore structure, Compressive modulus: up to ~200 kPa	Bone Tissue Engineering	[149]
Type I collagen from bovine Achilles tendon	-	nano-HAp	EDC/NHS	−20 °C	24 h	50–900 μm, Heteroporous morphology, Storage modulus: ~18 kPa	Bone Tissue Engineering	[167]
Type B gelatin from bovine skin	0.5% (*w*/*v*)	HAp	Acetone-water-based solution	−20 °C (24 h)	72 h	porosity 88%/85% 250/300 μm, -, Elastic modulus: ~4 MPa	Hard Tissue Engineering	[152]
Type I collagen from bovine tendon	-	GAG, BG	DHT and N-(3-dimethylaminopropyl)-N’-ethylcarbodiimide hydrochloride and NHS	-	24 h at −40 °C	porosity > 97%, -, Compressive modulus: up to ~5 kPa	Bone Tissue Engineering	[158]
Type I collagen from bovine dermis	-	GAG	DHT, EDC, and NHS	−60 °C to −10 °C	0 °C	55–243 μm, Aligned pores, -	Tendon Tissue Engineering	[168]
Type I collagen from bovine	0.5–1%	GAG	EDC and NHS	−10 °C and −40 °C	18 h	137 μm, Homogenous pore structure, Compressive modulus: ~4 kPa Tensile modulus: ~0.6 MPa	Tissue Engineering	[66]
Type I collagen from bovine	0.5% (*w*/*v*)	GAG	DHT	−70 °C to −10 °C	17 h at 0 °C	85–325 μm, -, -	Tissue Engineering	[159]
Collagen from young rats’ tail tendons	1%	CH, SF	EDC/NHS	−80 °C	48 h	200 μm, -, Young’s modulus: ~32 kPa	Bone Tissue Engineering	[169]
Type A gelatin from pig skin	2%	CH	GA	−190 °C or −27 °C	18 h at −55 °C	119–196 μm, Homogenous pore structure, -	Skin Regeneration	[69]
Type I collagen from bovine tendon	-	nano-CE	GNP	−80 °C	24–48 h	90–140 μm, Uniform pore structure, Young’s modulus: ~1.5 MPa	Wound Dressing	[170]
Gelatin from porcine skin	0.6% (*w*/*v*)	TCH-PLA	EDC and NHS	−20 °C (12 h)	24 h at −50 °C	261 μm, -, Compressive modulus: ~1 MPa Elastic modulus: ~1.75 MPa	Soft Tissue Engineering	[145]
Type I collagen from porcine	2% (*w*/*v*)	PLGA	EDC, NHS	−12 °C (4 h) and −80 °C (6 h)	48 h	porosity 99% 441/50 μm, -, Elastic modulus: ~9 kPa	Cartilage Tissue Engineering	[163]
Gelatin from porcine skin Collagen	3%	-	GA	−40 °C	12 h at −40 °C	porosity 95% 50–100 μm, -, -	Bone Tissue Engineering	[171]
Type I collagen from porcine	2% (*w*/*v*)	-	GA	−80 °C (6 h)	72 h	150–250 μm, Homogenous pore structure, Young’s modulus: ~20 kPa	Cartilage Tissue Engineering	[162]
Collagen from bovine Achilles tendon	1% (*w*/*w*)	-	Ethanol and 1-ethyl-3-(3-dimethylaminopropyl)-carbodiimide hydrochloride and NHS	−40 °C (2 h)	18 h	100 μm, -,-	Cancer Cell Invasion and Migration	[172]

## Data Availability

Not applicable.
